# New associations of serum β‐carotene, lycopene, and zeaxanthin concentrations with *NR1H3*, *APOB*, *RDH12*, AND *CYP* genes

**DOI:** 10.1002/fsn3.2705

**Published:** 2022-01-08

**Authors:** Ingrida Domarkienė, Asta Mažeikienė, Guostė Petrauskaitė, Zita Aušrelė Kučinskienė, Vaidutis Kučinskas

**Affiliations:** ^1^ Department of Human and Medical Genetics Faculty of Medicine Institute of Biomedical Sciences Vilnius University Vilnius Lithuania; ^2^ Department of Physiology Biochemistry, Microbiology and Laboratory Medicine Faculty of Medicine Institute of Biomedical Sciences Vilnius University Vilnius Lithuania

**Keywords:** association analysis, bioavailability, carotenoid concentration, genetic variation, single‐nucleotide polymorphisms (SNPs)

## Abstract

Variation in carotenoid bioavailability at individual and population levels might depend on host‐related factors where genetic variation has a part to play. It manifests itself through the proteins involved in carotenoid intestinal absorption and metabolism, blood lipoprotein transport, or tissue uptake. This study aims to identify novel SNPs which could be associated with carotenoid serum concentrations. A total of 265 self‐reported healthy individuals of Lithuanian origin were genotyped (Illumina HumanOmniExpress‐12v1.0 or v1.1 and Infinium OmniExpress‐24v1.2 arrays) and fasting blood serum concentrations of β‐ and α‐carotene, β‐cryptoxanthin, lycopene, lutein, and zeaxanthin were measured (Shimadzu Prominence HPLC system). According to the individual carotenoid concentrations, the cohort was subdivided into quartiles. Q1 and Q4 were used for the following association analysis. The set of 2883 SNPs in 109 potential candidate genes (assumed for a direct or indirect role in carotenoid bioavailability) was analyzed. Liver X receptor alpha (*NR1H3*) “transport” polymorphisms rs2279238 (*p* = 2.129 × 10^−5^) and rs11039155 (*p* = 2.984 × 10^−5^), and apolipoprotein B (*APOB*) “transport” polymorphism rs550619 (*p* = 4.844 × 10^−5^) were associated with higher zeaxanthin concentration. Retinol dehydrogenase 12 (*RDH12*) “functional partner” polymorphism rs756473 (*p* = 7.422 × 10^−5^) was associated with higher lycopene concentration. Twenty‐one cytochrome P450 (*CYP2C9*, *CYP2C18,* and *CYP2C19*) “metabolism” polymorphisms in locus 10q23.33 were significantly associated with higher β‐carotene concentration. To conclude, four novel genomic loci were found to be associated with carotenoid serum levels. Zeaxanthin, lycopene, and β‐carotene serum concentrations might depend on genetic variation in *NR1H3*, *APOB*, *RDH12* and *CYP2C9, CYP2C18,* and *CYP2C19* genes.

## Graphical abstract

DNA samples of 265 self‐reported healthy individuals of Lithuanian ethnicity were genotyped using genome‐wide microarrays. Blood serum concentrations of β‐ and α‐carotene, β‐cryptoxanthin, lycopene, lutein, and zeaxanthin were measured by the high‐performance liquid chromatography method. Carotenoid concentrations were used to group individuals into comparable groups (quartile Q1 and Q4 groups) for the following association analysis. A targeted set of 2883 SNPs, representing 109 potential candidate genes (assumed for direct or indirect role in the carotenoid bioavailability), was analyzed. Twenty‐five SNPs were found to be associated with carotenoid serum levels. Zeaxanthin, lycopene, and β‐carotene serum concentrations might depend on genetic variation in NR1H3, APOB, RDH12 and CYP2C9, CYP2C18, and CYP2C19 genes.

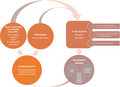

## 1 INTRODUCTION

Carotenoids found at the highest concentrations in human blood are the same as those commonly found in food products—β‐carotene, lycopene, lutein, β‐cryptoxanthin, α‐carotene, and zeaxanthin (Khachik et al., 1992).

Total and individual consumption of carotenoids varies greatly between and within populations, and this is mainly reflected in the consumption of fruits and vegetables. Bioavailability is also a subject of variation. It depends on the processes of bioaccessibility, absorption, tissue distribution, turnover, and excretion or the possible interactions of individual carotenoids. However, the underlying mechanisms are still poorly understood (Bohn et al., 2017). All those processes may be influenced by many factors including diet (e.g., food matrix, fat); host‐related factors such as gender, age, medical conditions (e.g., HIV, hyperthyroidism); lifestyle (e.g., physical activity, smoking), and genetic factors. Studies have shown that interindividual variability in the bioavailability of carotenoids can be modulated by single‐nucleotide polymorphisms (SNPs) (Borel et al., 2011, 2014, 2015a, 2015b; Lietz et al., 2012; Merle et al., 2013). These belong to the genes involved in the intestinal uptake or efflux of carotenoids as well as carotenoid metabolism and transport.

The general metabolism of carotenoids involves the following: (1) release from the food matrix, (2) solubilization into mixed micelles, (3) uptake by intestinal cells, (4) incorporation into chylomicrons or high‐density lipoprotein (HDL), (5) secretion into the lymph and circulation, and (6) tissue uptake and retention (Shmarakov et al., 2013). As nicely summarized by Bohn et al. (2017), the proteins and their function in the aforementioned metabolic processes are determined by certain genes and their variation. There is quite a list of already‐known or speculated SNPs that affect carotenoid metabolism. They are in genes related to digestion (*PNLIP*, *CLPS*, and *LIPS*), absorption (*SCARB1*, *CD36*, *NPC1L1*, *ABCG5, ABCG8, ABCG2*, *ABCA1*, *ABCB1*, and *ISX*), intracellular cleavage (*BCO1* and *BCO2*), intracellular transport and other functions (*ELOVL2*, *INSIG2*, *I‐FABP*, and *SLC27A6*), chylomicron secretion (*MTP*), blood, liver metabolism, lipoprotein distribution (*LPL*, *APOA1*, *APOA4*, *APOE*, *APOB*, *LDLR*, *LIPC*, *CYP26B1*, and *CETP*), tissue incorporation (*GSTP1*, *STARD3*, and *RPE65*), and other complex processes such as obesity or insulin metabolism (*MTTP*, *MPOD*, *SLIT3*, *DHRS2*, *SOD2*, *MC4R*, *COBLL1*, *CXCL8*, *TCF7L2*, *PKD1L2*, *IRS1*, and *SETD7*) (Bohn et al., 2017; Moran et al., 2018). Various proteins participate in carotenoid metabolic pathways but not all of them are known yet. Despite the overwhelming success of association studies in general, only some studies have analyzed carotenoid concentrations (Buniello et al., 2019). Most of them were performed in populations of mixed ancestry (admixed populations) and there is a growing demand for such studies in distinct or ethnic populations (Sirugo et al., 2019) to replenish current knowledge of associated genetic factors. This study aimed to search for new genetic loci associated with serum carotenoid concentrations in the Lithuanian population cohort.

## 2 MATERIAL AND METHODS

### 2.1 Study cohort

A total of 265 self‐reported healthy genetically unrelated individuals (131 men aged 50 ± 10 years and 134 women aged 49 ± 9 years) from the Lithuanian population (six ethnolinguistic regions) with at least three generations living in Lithuania were recruited for this study. As it was part of the LITGEN project (Lithuanian Population Genetic Diversity and Structure Variations Associated with the Evolution and Most Common, Prevalent Diseases), which aimed to analyze the genomic structure of the Lithuanian population, this study had several inclusion criteria: (1) study participants had to be of Lithuanian origin; (2) families of all participants must have been living in their ethnolinguistic region for at least three generations. We did not include individuals who were not of Lithuanian origin. The study was approved by the Vilnius Regional Biomedical Research Ethics Committee (No. 158200‐05‐329‐79). Informed written consent from all participants was obtained.

### 2.2 Collection of blood, sample preparation, storage, and transportation

Study individuals had to arrive at the primary healthcare center located in the city or district they lived from 7:30 to 10:00 a.m. in fasting condition for at least 12 h, abstaining from smoking, alcohol, and medications. In the facility, venous blood samples were taken for serum carotenoid analysis (5 ml BD Vacutainer^®^ SST II Advance tubes; Becton Dickinson) and DNA extraction (10 ml BD Vacutainer^®^ K2EDTA tubes; Becton Dickinson).

For serum sample preparation, after 40 min, tubes with blood samples were centrifuged for 10 min at 1150 *g* (centrifuge LMC‐3000, Biosan). Samples were stored at +2 to +8°C and transported in thermostable containers (polystyrene foam boxes) to avoid temperature variations. Within 3–6 h, serum samples were transported to the Centre of Laboratory Medicine of the Santaros Clinics, Vilnius University Hospital and deep‐frozen at −80°C. Carotenoid concentration was analyzed within 6–12 months after the sample collection. Sample collection, preparation, transportation, and all further stages of the analysis were carried out in such way as to avoid direct sunlight or intensive artificial light in order to prevent the dissociation or isomerization of carotenoids.

To prepare samples for genotyping analysis, genomic DNA was extracted from venous blood. There were two methods of genomic DNA extraction: either phenol‐chloroform (according to the approved laboratory protocol) or using magnetic beads (DNA extraction technical note: MagneSil^®^ Genomic, Large Volume System, Instructions for Use of Products A4080, A4082, A4085 Technical Bulletin) on the automatic robotic system *TECAN Freedom EVO^®^ 200* (manufacturer *TecanSchweiz AG,* CH).

The concentration and purity of extracted genomic DNA were measured with a NanoDrop^®^ spectrophotometer (NanoDrop^®^ Technologies Inc.) following the NanoDrop^®^ ND‐1000 Spectrophotometer User Manual. DNA purity values were ~1.8. The genomic DNA concentration used for genotyping was ~50 ng/µl.

### 2.3 Genotyping

The genotyping was performed on the Illumina HiScan™SQ instrument (Illumina Inc.) using the Illumina Infinium^®^ HD SNP assays HumanOmniExpress‐12 v1.0, HumanOmniExpress‐12 v1.1, and Infinium OmniExpress‐24 v1.2.

Genotyping was performed according to the protocols provided by the manufacturer: Infinium^®^ HD Assay Ultra Manual Experienced User Card, Infinium^®^ HD Assay Ultra Protocol Guide, Illumina Infinium^®^ Assay Lab Set‐Up and Procedures.

The genotyping, using Illumina Infinium^®^ HD SNP assay HumanOmniExpress‐12 v1.0, was performed at the Tartu University Institute of Molecular and Cell Biology (Estonia) according to the Infinium^®^ Multi‐Use Assay, Manual Protocol, 15013850 Rev.

### 2.4 Serum carotenoid extraction and analysis

Concentrations of serum β‐carotene, α‐carotene, β‐cryptoxanthin, lycopene, lutein, and zeaxanthin were analyzed using the high‐performance liquid chromatography (HPLC) method according to the slightly modified Boehm et al. methodology (Fröhlich et al., 2006).

For carotenoid extraction, a microtube with 1 ml of serum and 500 μl of an ethanolic echinenone (β,β‐carotene‐4‐one; Chromadex) solution (used as internal standard) was vortexed for 30 s. Then, 400 µl of hexane with 0.1% of butylhydroxytoluene was added and vortexed for 1 min. The sample was subsequently centrifuged at 12 755 *g* for 2 min. The extraction steps were repeated twice for each sample. The supernatant (hexane layer) was transferred after each centrifugation into the microtube and finally completely dried, applying a gentle flow of the nitrogen gas at a temperature of 30°C ± 1°C. The residue was dissolved in 250 μl of methanol (MeOH; Sigma‐Aldrich) and methyl‐tert‐butyl ether (MTBE; Carl Roth) mixture (1:1 v/v) and centrifuged for 4 min at 12 755 *g* and then transferred into a chromatographic bottle.

Carotenoid concentrations were detected using the following carotenoid standards (crystal form) for HPLC, purity 95%–98% (Chromadex): β‐carotene (β, β‐carotene) 96%, β‐cryptoxanthin ((3*R*)–β, β‐carotene‐3‐ol) 97%, α‐carotene ((6′R)‐β, ε‐carotene) 97%, echinenone (β, β‐carotene‐4‐one) 98%, lutein (xanthophyll (3*R*,3′*R*,6′*R*)–β, ε‐carotene‐3,3′‐diol) 96%, lycopene (ψψ‐carotene) 95%, zeaxanthin ((3*R*,3′*R*)‐β, β‐carotene‐3,3′‐diol) 97%, dissolved ether in a toluene–cyclohexane mixture (1:4 v/v), or in ethanol (1:10).

Chromatographic separation was performed using an HPLC system (Shimadzu Prominence) with C30 (250 mm × 4.6 mm; particle size 5 μm) HPLC column (Dr. Maisch GmbH) and guard pre‐columns (C30, 20 mm × 4.6 mm). The column temperature was 23°C ± 1°C and the injection volume was 30 μl. Carotenoids were separated with a two‐component mobile phase of MeOH (Eluent A) and MTBE (Eluent B). Flow rate was set at 1.3 ± 1 ml/min and the gradient elution was as follows: 10% Eluent B (initial), 10%–45% Eluent B (0.01–34.0 min), isocratic 60% Eluent B (35.0–44.0 min), and 60%–10% Eluent B (45.0–50.0 min). A UV–visible spectrophotometer was used for detection with wavelength 450 nm (470 nm for lycopene concentration measurements). The identification of each compound was confirmed using retention time and UV spectra of the pure compounds. For all carotenoids, the concentrations are reported in micromoles per liter (µmol/L) of serum. At least two blood serum samples were tested for carotenoid concentrations for each study individual. The reproducibility of the internal standard was 92% ± 12%. Carotenoids were quantified using external calibration curves. Six concentrations of calibrator mixture were prepared for α‐carotene, β‐carotene, β‐cryptoxanthin, lutein, zeaxanthin, and lycopene by diluting the carotenoid standard working solutions in a mixture of MeOH and MTBE (1 + 1, v/v) in proportions of 1:10, 1:20, 1:50, 1:100, 1:250, and 1:500. The calibration curve of each carotenoid was made by plotting the peak area of the analyte against the concentration of the analyte. Correlation coefficients (*R* ^2^) for α‐carotene, β‐carotene, β‐cryptoxanthin, lutein, zeaxanthin, and lycopene were not lower than 0.995. The amounts of carotenoids were calculated from the regression equations. Duplicate analyses were carried out and the data were expressed as mean ± standard deviation.

### 2.5 Set of genes

Genes for the analysis were compiled into three groups based on a shared biological or functional property related to carotenoid bioavailability. The set of genes and SNPs in detail is provided in Table S1.

The first group consisted of 43 genes (1040 SNP markers) encoding proteins related (or possibly related) to carotenoid uptake, distribution, metabolism, and excretion, for instance, digestion enzymes fostering micellization (*PNLIP*), uptake/efflux transporters (*SCARB1, CD36, NPC1L1*), intracellular transporters (*FABP2*), those participating in the processes of secretion into chylomicrons (*APOB, MTTP*), carotenoid metabolism in blood and liver (*LPL*, *APOE, LDLR*), and distribution to target tissues such as adipose tissue or the macula (*GSTP1, STARD3*).

The second group consisted of 37 genes (1073 SNP markers) encoding proteins responsible for intracellular carotenoid cleavage (*BCO1, BCO2*) and their functional partners or transcription factors.

The third group consisted of 29 genes (770 SNP markers) encoding cytochrome P450 enzymes that are related to retinol metabolism (according to The Human Protein Atlas (Uhlen et al., 2015)).

### 2.6 Statistics

Descriptive statistics for serum carotenoid concentrations were calculated by open‐source software RStudio (RStudio Team, 2020). According to the carotenoid concentrations, individuals were subdivided into quartiles. Quartiles Q1 and Q4 were used for the following association analysis.

Genotyping data quality control (QC) and association analysis were performed using the PLINK whole‐genome association analysis toolset (PLINK v1.90b (Chang et al., 2015)). SNPs included in the association analysis met the following genotyping data quality control criteria (Anderson et al., 2010): minor allele frequency (MAF) > 0.05; missingness per marker (GENO) < 0.01; the Hardy–Weinberg equilibrium test's *p*‐value >.001 (chi‐squared test); missingness per individual (MIND) < 0.05. The chi‐squared statistic was used for association analysis to evaluate differences in allele frequencies in each SNP between the Q1 and Q4 quartiles. Significant SNPs were provided with the odds ratio (OR) and 95% confidence interval (95% CI) calculations. The Bonferroni adjustment (α = 0.05/N) and adaptive permutation procedure were performed for multiple comparisons. The significance level for the analysis was set according to the adaptive permutation recommendations (Che et al., 2014). The power of the test values, according to the different sample size groups, was 0.77–0.79 and was calculated using post hoc calculation with the G*Power 3.1.9.4 tool designed by Franz Faul, University of Kiel, Germany (Faul et al., 2007).

## 3 RESULTS

The characteristics (age, gender, body mass index (BMI)) of the study cohort of 265 individuals are shown in Table 1. Mean values of age and BMI were similar in both gender groups. According to the BMI values, the study cohort could be classified as overweight.

**TABLE 1:** Main characteristics of the study cohort

Characteristic	Men			Women		
	*N* = 131			*N* = 134		
	Median	Mean	SD	Median	Mean	SD
Age, years	48	50	10	46	49	9
BMI, kg/m^2^	28	29	4	27	28	5

Abbreviations: BMI, body mass index; *N*, number of individuals; SD, standard deviation.

Based on the questionnaire sociodemographic data, it was found that the majority of studied individuals resided in an urban setting (61%); 85% of the study cohort indicated as having special secondary or higher education and the majority of participants worked as employees (31%) or clerks (46%). The study cohort was not characterized by high physical activity; 43% of individuals had lifestyles that are not physically active and 47% of individuals had lifestyles involving small physical activity, including walking and cycling to and from work, easy gardening, fishing, etc.

The predominant blood serum carotenoid in the study cohort as well as among the group of men was found to be lycopene (33% and 36%, respectively) while among women, it was β‐carotene (34%). Zeaxanthin concentration was the lowest in all groups (1%). Mean and median values of the analyzed carotenoids in the study cohort are reported in Table 2.

**TABLE 2:** Analyzed blood serum carotenoid concentrations (μmol/L)

	Men (*N* = 131)		Women (*N* = 134)		Total (*N* = 265)	
	Mean ± SD	Median	Mean ± SD	Median	Mean ± SD	Median
Lycopene	0.58 ± 0.34	0.495	0.52 ± 0.28	0.473	0.55 ± 0.32	0.487
α‐Carotene	0.13 ± 0.08	0.112	0.19 ± 0.14	0.151	0.16 ± 0.12	0.126
β‐Carotene	0.41 ± 0.31	0.350	0.61 ± 0.42	0.525	0.51 ± 0.38	0.414
β‐Cryptoxanthin	0.10 ± 0.07	0.083	0.16 ± 0.15	0.117	0.13 ± 0.12	0.095
Lutein	0.30 ± 0.15	0.268	0.27 ± 0.15	0.242	0.28 ± 0.15	0.263
Zeaxanthin	0.01 ± 0.01	0.008	0.01 ± 0.02	0.007	0.01 ± 0.02	0.008
Total carotenoid conc.	1.59 ± 0.75	1.529	1.82 ± 0.82	1.707	1.65 ± 0.77	1.592

Abbreviations: *N*, number of individuals; SD, standard deviation.

To perform association analysis, carotenoid concentrations were subdivided into quartiles to form groups of samples to be compared (Table 3).

**TABLE 3:** Analyzed carotenoid concentration quartiles

Quartile	Lyc	*N*	α‐car	*N*	β‐car	*N*	β‐crypto	*N*	Lut	*N*	Zeax	*N*
	Concentration (μmol/L)											
Q1	≤0.323	67	≤0.077	69	≤0.245	67	≤0.060	68	≤0.182	67	≤0.005	74
Q2	0.323–0.487	67	0.077–0.126	65	0.245–0.414	66	0.061–0.095	66	0.182–0.263	66	0.005–0.008	59
Q3	0.487–0.734	65	0.126–0.206	65	0.414–0.681	66	0.095–0.165	65	0.2632–0.354	66	0.008–0.012	67
Q4	>0.734	66	>0.206	66	>0.681	66	>0.165	66	>0.354	66	>0.012	65

Abbreviations: Lut, Lutein; Lyc, Lycopene; *N*, number of samples; Zeax, Zeaxanthin; α‐car, α‐carotene; β‐car, β‐carotene; β‐crypto, β‐cryptoxanthin.

**FIGURE 1:**
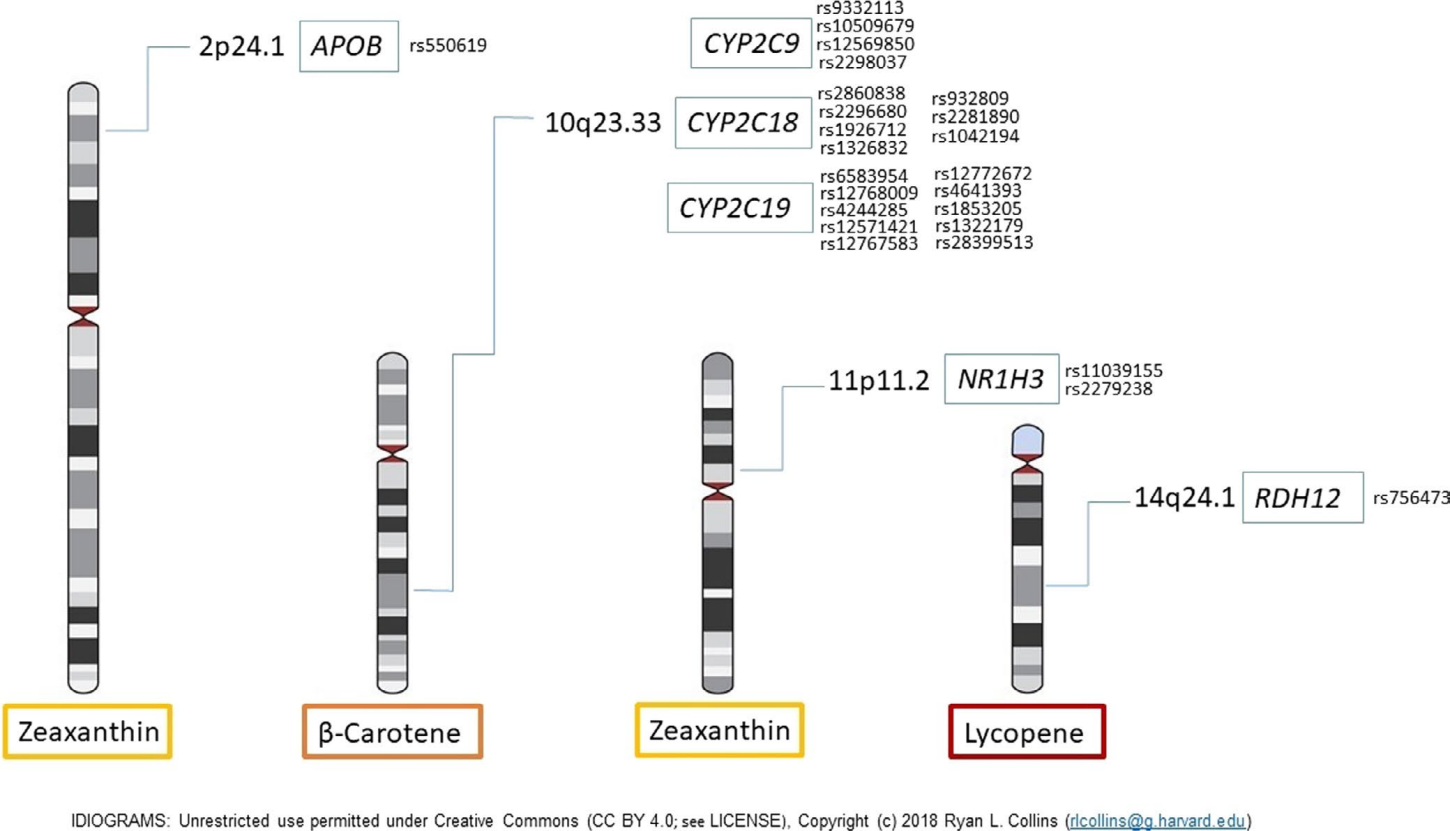
Significant new associations of zeaxanthin and SNPs in 2p24.1, 11p11.2 loci, β‐carotene and SNPs in 10q23.33 locus, and lycopene and SNP in 14q24.1 locus. *APOB—*apolipoprotein B, *NR1H3—*nuclear receptor subfamily 1 group H member 3, *RDH12—*retinol dehydrogenase 12, *CYP2C9—*cytochrome P450 family 2 subfamily C member 9, *CYP2C19—*cytochrome P450 family 2 subfamily C member 19, *CYP2C18—*cytochrome P450 family 2 subfamily C member 18, *CYP2C19—*cytochrome P450 family 2 subfamily C member 1

Before association analysis, data quality control (procedure described in Section 2.6) of 265 genotyped samples and 2883 SNPs was performed. All samples passed the 5% threshold of the missing genotypes rate. One hundred and fifty‐six SNPs did not pass the 1% missingness rate, one SNP deviated from the Hardy–Weinberg equilibrium, and 973 SNPs with rare alleles of frequency <5% were excluded from further analysis. Finally, 1 753 SNPs (gene group I had 710, group II 661, and group III 382 SNPs) and 256 samples were set for subsequent association analysis. After association analysis of sets of SNP markers in the two groups of individuals subdivided according to the carotenoid concentrations (quartiles Q1 and Q4), we found new genetic loci associated with the serum carotenoid concentrations. Significant SNPs along with the chi‐squared, p‐values, and OR with 95% CI are presented in Table S2. Significant associations are depicted in Figure 1.

From analyzing the association of the serum carotenoid concentrations and SNPs of the first gene group, we found SNP rs550619 of the *APOB* gene to be significantly associated (*p* = 4.844 × 10^−5^) with blood serum zeaxanthin concentration. Minor allele G is related to the higher blood serum zeaxanthin concentration. The SNP is located in intron 5 (out of 28) of the *APOB* gene encoding apolipoprotein B.

Analyzing the second gene group, we found three SNPs to be significantly associated with blood serum carotenoid concentration. SNPs rs11039155 (*p* = 2.984 × 10^−5^) and rs2279238 (*p* = 2.129 × 10^−5^) of the *NR1H3* gene were associated with higher zeaxanthin concentration, while SNP rs756473 (*p* = 7.422 × 10^−5^) of the *RDH12* gene was associated with higher lycopene concentration. Intronic variant rs11039155 and synonymous variant rs2279238 are both located in the *NR1H3* gene encoding nuclear receptor subfamily 1 group H member 3. SNP rs756473 is located in intron 3 (out of 8) of the *RDH12* gene encoding retinol dehydrogenase 12.

Analyzing the third gene group, we found 21 SNPs of the locus 10q23.33 encompassing genes *CYP2C9*, *CYP2C18*, and *CYP2C19* to be significantly associated with the higher β‐carotene concentration. The majority of associated SNPs are intronic except 3′UTR variant rs1042194 in the *CYP2C18* gene and synonymous variant rs4244285 in the *CYP2C19* gene.

The serum α‐carotene, β‐cryptoxanthin, and lutein concentrations were not associated with any of the studied genetic markers (SNPs).

## 4 DISCUSSION

The results of blood serum carotenoid mean concentrations can be assessed in the context of data from analogous studies in other countries. Both total concentrations of carotenoids and concentrations of individual carotenoids in the Lithuanian study group were among the lowest compared with the results of a cross‐sectional study of a cohort of 45‐ to 65‐year olds carried out in 16 regions of nine European countries (Al‐Delaimy et al., 2004). However, the established concentrations of total and individual carotenoids in Lithuanians are higher than in healthy American, Chinese, and Korean adults (Yeum et al., 1999). The predominant blood serum carotenoid in our study cohort was lycopene, and zeaxanthin concentration was the lowest. These findings are in line with the results presented in the study mentioned above of other European countries (Al‐Delaimy et al., 2004) where lycopene was quantitatively a predominant carotenoid, followed by β‐carotene, while α‐carotene and zeaxanthin levels were the lowest. Lycopene and β‐carotene were major carotenoids in American individuals, whereas lutein was the predominant carotenoid in the Chinese (Yeum et al., 1999). Moreover, serum carotenoid concentrations differed across ethnic groups (Sanchez et al., 2021). These findings suggest that population‐specific variability might significantly predict total and individual blood carotenoid levels in different geographical regions.

This study aimed to identify new SNPs and genes related to serum carotenoid concentrations. One of the objectives was to complement the current knowledge on genetic factors contributing to interindividual differences of carotenoid quantities found in the blood. The association analysis involved the sets of SNPs of genes encoding: (1) proteins taking part in carotenoid absorption, metabolism, and transport; (2) proteins that are transcription factors or functional partners of well‐known participants involved in carotenoid metabolism; and (3) proteins involved in carotenoid catabolism and excretion—cytochrome P450 enzymes. Our results showed new genetic loci significantly related to serum zeaxanthin, lycopene, and β‐carotene concentrations in the studied cohort of the general Lithuanian population.

Zeaxanthin was associated with the *APOB* and *NR1H3* genes. *APOB* encoding apolipoprotein B occurs in plasma in two isoforms: apoB‐100 and apoB‐48. apoB‐48 is the main apolipoprotein constituent of chylomicrons while apoB‐100 is a component of LDL and VLDL. The majority (55%) of carotenoids are transported as the circulating form of LDL components, while zeaxanthin involves LDL and HDL (Shmarakov et al., 2013). Studies have shown significant associations of SNPs in the *APOB* gene with β‐carotene (Borel et al., 2007, 2015a), lycopene (Borel et al., 2007, 2015b), and lutein (Borel et al., 2014) bioavailability and concentration in the blood. For the first time, we report that *APOB* SNP (rs550619) is found to be significantly associated with zeaxanthin serum concentration. The minor allele (G) of the SNP is related to the higher blood serum zeaxanthin concentration (OR 7.3) in the studied Lithuanian population. Our findings correspond with others claiming that SNPs in the *APOB* gene may modulate carotenoid blood concentration, indicating that chylomicron assembly and lipoprotein clearance are important factors in determining carotenoid blood status.

The other gene associated with zeaxanthin concentration was *NR1H3*. Our study showed that minor alleles of the SNPs rs11039155 and rs2279238 were related to the higher zeaxanthin blood serum concentration (ORs 3.5 and 3.4, respectively). The *NR1H3* gene was never previously associated with carotenoid blood levels. *NR1H3* codes for nuclear receptor subfamily 1 group H member 3 also known as oxysterols receptor LXR‐alpha. The protein forms a heterodimer with retinoid X receptor (RXR) and regulates the expression of target genes containing retinoid response elements. Alternatively, spliced *NR1H3* gene transcripts translated to the different isoforms of the protein have been found. Studies suggest that NR1H3 plays an important role in the regulation of cholesterol homeostasis (Edwards et al., 2002) and increased expression of the ATP binding cassette transporters ABCA1, ABCG1, and apolipoprotein E (apoE), all of which participate in the transfer of intracellular and plasma membrane cholesterol to HDL (Costet et al., 2000; Laffitte et al., 2001). Our findings suggest that NR1H3 may indirectly participate in carotenoid metabolism, which is closely related to lipid metabolism and may modulate carotenoid transport dependent on lipoprotein assembly.

Lycopene was associated with the genetic marker in the *RDH12* gene encoding retinol dehydrogenase 12. RDH12 is highly expressed in photoreceptors and catalyzes the oxidation and reduction in both all‐*trans*‐ and *cis*‐retinal in the presence of NADP+or NADPH as cofactors (Belyaeva et al., 2005; Haeseleer et al., 2002). Retinal is a form of vitamin A and may be produced from provitamin A carotenoids: α‐carotene, β‐carotene, or β‐cryptoxanthin but not from non‐provitamin A carotenoid such as lycopene. Even though lycopene is not accumulated in the retina, lycopene isomers were found in the eye structures (Khachik et al., 2002). Common localization of lycopene and RDH12 suggests the assumption of indirect interaction. Thus, the association with *RDH12* raises many questions and requires more detailed research.

Finally, β‐carotene was found to be associated with 21 SNPs located in three cytochrome P450 genes: *CYP2C9*, *CYP2C18,* and *CYP2C19*. The cytochrome P450s are a superfamily of enzymes that catalyze the metabolism of xenobiotic drugs and environmental chemicals as well as many endogenous compounds. The human CYP2C subfamily consists of four members—CYP2C18, CYP2C19, CYP2C9, and CYP2C8—and are found predominantly in the liver where they comprise ~20% of the total cytochrome P450 (Chen & Goldstein, 2009). Some of the CYP450 enzymes are involved in metabolism and possibly catabolism of the biologically active vitamin A metabolite—retinoic acid (RA). It was shown that CYP2C9, CYP2C18, and CYP2C19 catalyze the hydroxylation of RA (Marill et al., 2000; Nadin & Murray, 1999). β‐Carotene is a pro‐vitamin A carotenoid and can be metabolized into RA; thus, our finding suggests that genetic variation in the CYP2C subfamily enzymes' coding genes may impact β‐carotene serum level.

It should not be forgotten that even though SNPs represent more than 96% of variation (Genomes Project C et al., 2015), other genetic variations such as copy number variants, deletions and/or insertions of several nucleotides, as well as epigenetic modifications are also present and might have an effect. We should therefore consider all of the genetic variations that can have a significant impact on carotenoid concentrations. It is also crucial to perform association studies in different populations to find out population‐specific variation.

Each population may have some exceptional genetic characteristics and association studies may fail to replicate these. Thus, replication studies should focus on populations with genetic ancestry similar to the tested population. Lithuanians were found to be homogenous and genetically close to neighboring populations, for example, Slavs (Russians and Poles) and Finno‐Ugrians (Estonians and Finns) (Kasperavičiūtė et al., 2004). However, it was confirmed that the Lithuanian population shows genetic distinctiveness from other European countries as well (Urnikyte et al., 2019). This was demonstrated by genetic structure, divergence time, and positive natural selection analysis. The latter, interestingly, showed that among the top signatures of positive selection found are candidate regions associated with diet. This is one of the reasons why the Lithuanian population is unique and why some genetic associations found in other studies do not reproduce.

As for the study limitations, we denote that the study cohort was extracted from the representative cohort of the Lithuanian population subdivided into six ethnolinguistic regions of Lithuania ( Jakaitienė & Kučinskas, 2013). Despite potential interest in the precursors of carotenes and their biological effects, the main goal of the study was to broaden the knowledge of genetic variation related to interindividual variability in the bioavailability and metabolism of carotenoids, which are found at the highest concentrations in human blood, are the most abundant in food products, and are linked to health benefits in epidemiological studies. We also state that generalization of the present findings should be viewed taking into account that lycopene intake was found to be among the lowest in the Lithuanian population compared with other European counties (Mažeikienė et al., 2015), and assessment of total carotenoid consumption among Lithuanians is lacking. Additional studies should be performed to differentiate the importance of nutritional habits and genetic variability on serum carotenoid concentrations.

## 5 CONCLUSIONS

In conclusion, this study identified four new loci associated with zeaxanthin, lycopene, or β‐carotene serum concentrations. Our findings reproduce the idea that carotenoid bioavailability depends on genetic variation. We also note that some associations might be population specific as performed in the ethnic Lithuanian population. The role of associated genes in carotenoid metabolism is not characterized well. However, our significant results contribute to the current science with new insights into interindividual variation in carotenoid bioavailability and raise further questions to be answered.

## CONFLICT OF INTEREST

All authors declare no conflicting interests.

## AUTHOR CONTRIBUTIONS

**Ingrida Domarkienė:** conceptualization (equal) ; dataCuration(equal);investigation (equal); methodology (equal); supervision (equal);writingOriginalDraft (equal); writingReviewEditing (lead). **Asta Mažeikienė:** conceptualization (equal) ; dataCuration (equal); investigation (equal); methodology (equal); supervision (equal);writingOriginalDraft (equal); writingReviewEditing (equal). **Guostė Petrauskaitė:** formalAnalysis (lead) ; visualization (lead); writingOriginalDraft (equal); writingReviewEditing (equal). **Zita Aušrelė Kučinskienė:** dataCuration (lead) ; supervision (equal); writingReviewEditing (supporting). **Vaidutis Kučinskas:** dataCuration (lead) ; fundingAcquisition (lead); supervision (equal); writingReviewEditing (supporting).

## Supporting information

Table S1

Table S2

## Data Availability

All necesary data is provided in the article. Nevertheless authors agree to share raw data upon request.
