# Supplementary material for: New associations of serum β‐carotene, lycopene, and zeaxanthin concentrations with NR1H3, APOB, RDH12, AND CYP genes
Source: Food Sci Nutr. 2022 Jan 8;10(3):763–71. doi: 10.1002/fsn3.2705 (PMC8907718; doi:10.1002/fsn3.2705)
Supplement: Supplementary file 1 — Table S1 [file FSN3-10-763-s002.docx]

Supplemental Table 1. The set of genes selected for the association analysis.

| **Group I. Genes related to carotenoid uptake, transport, metabolism, excretion, and distribution** | |
| --- | --- |
| **Gene symbol** | **Gene name** |
| *ABCA1* | ATP Binding Cassette Subfamily A Member 1 |
| *ABCB1* | ATP Binding Cassette Subfamily B Member 1 |
| *ABCG5* | ATP Binding Cassette Subfamily G Member 5 |
| *ABCG8* | ATP Binding Cassette Subfamily G Member 8 |
| *APOA1* | Apolipoprotein A1 |
| *APOA4* | Apolipoprotein A4 |
| *APOB* | Apolipoprotein B |
| *APOE* | Apolipoprotein E |
| *CD36* | CD36 Molecule |
| *CETP* | Cholesteryl Ester Transfer Protein |
| *CXCL8/IL8* | C-X-C Motif Chemokine Ligand 8 |
| *ELOVL2* | Elongation of Very Long Chain Fatty Acids Like 2 |
| *FABP1* | Fatty Acid Binding Protein 1 |
| *FABP2* | Fatty Acid Binding Protein 2 |
| *GPIHBP1* | Glycosylphosphatidylinositol Anchored High Density Lipoprotein Binding Protein 1 |
| *GSTP1* | Glutathione S-Transferase Pi 1 |
| *HSPG2* | Heparan Sulfate Proteoglycan 2 |
| *INSIG2* | Insulin Induced Gene 2 |
| *LCAT* | Lecithin-Cholesterol Acyltransferase |
| *LDLR* | Low Density Lipoprotein Receptor |
| *LIPC* | Lipase C, Hepatic Type |
| *LPL* | Lipoprotein Lipase |
| *LRP1* | LDL Receptor Related Protein 1 |
| *MTTP* | Microsomal Triglyceride Transfer Protein |
| *NPC1* | NPC Intracellular Cholesterol Transporter 1 |
| *NPC1L1* | NPC1 Like Intracellular Cholesterol Transporter 1 |
| *NR4A1* | Nuclear Receptor Subfamily 4 Group A Member 1 |
| *NR4A2* | Nuclear Receptor Subfamily 4 Group A Member 2 |
| *NR4A3* | Nuclear Receptor Subfamily 4 Group A Member 3 |
| *PKD1L2* | Polycystic Kidney Disease Protein 1-Like 2 |
| *PNLIP* | Pancreatic Lipase |
| *RBP1* | Retinol Binding Protein 1 |
| *RBP2* | Retinol Binding Protein 2 |
| *RBP4* | Retinol Binding Protein 4 |
| *RPE65* | Retinal Pigment Epithelium-Specific 65 KDa Protein |
| *SAR1B* | Secretion Associated Ras Related GTPase 1B |
| *SCARB1* | Scavenger Receptor Class B Member 1 |
| *SLC27A6* | Solute Carrier Family 27 Member 6 |
| *SOD2* | Superoxide Dismutase 2 |
| *STARD3* | StAR Related Lipid Transfer Domain Containing 3 |
| *STRA6* | Signaling Receptor and Transporter of Retinol STRA6 |
| *TCF7L2* | Transcription Factor 7 Like 2 |
| *TTR* | Transthyretin |
| **Group II. Genes related to carotenoid intracellular cleavage and their functional partners or gene expression regulators** | |
| *ABLIM1* | Actin Binding LIM Protein 1 |
| *AKR1C3* | Aldo-Keto Reductase Family 1 Member C3 |
| *ALDH1A1* | Aldehyde Dehydrogenase 1 Family Member A1 |
| *ALDH1A2* | Aldehyde Dehydrogenase 1 Family Member A2 |
| *ALDH1A3* | Aldehyde Dehydrogenase 1 Family Member A3 |
| *ALOX15* | Arachidonate 15-Lipoxygenase |
| *AOX1* | Aldehyde Oxidase 1 |
| *BCO1* | Beta-Carotene Oxygenase 1 |
| *BCO2* | Beta-Carotene Oxygenase 2 |
| *CDO1* | Cysteine Dioxygenase Type 1 |
| *DHRS3* | Dehydrogenase/Reductase 3 |
| *DHRS4* | Dehydrogenase/Reductase 4 |
| *DHRS9* | Dehydrogenase/Reductase 9 |
| *GATA6* | GATA Binding Protein 6 |
| *HNF4A* | Hepatocyte Nuclear Factor 4 Alpha |
| *HSD17B1* | Hydroxysteroid 17-Beta Dehydrogenase 1 |
| *ISX* | Intestine Specific Homeobox |
| *LRAT* | Lecithin Retinol Acyltransferase |
| *MEF2* | Myocyte Enhancer Factor 2A |
| *NR1H2* | Nuclear Receptor Subfamily 1 Group H Member 2 |
| *NR1H3* | Nuclear Receptor Subfamily 1 Group H Member 3 |
| *POU2F1* | POU Domain, Class 2, Transcription Factor 1 |
| *PPARG* | Peroxisome Proliferator Activated Receptor Gamma |
| *RARA* | Retinoic Acid Receptor Alpha |
| *RBP2* | Retinol Binding Protein 2 |
| *RDH10* | Retinol Dehydrogenase 10 |
| *RDH11* | Retinol Dehydrogenase 11 |
| *RDH12* | Retinol Dehydrogenase 12 |
| *RDH13* | Retinol Dehydrogenase 13 |
| *RDH14* | Retinol Dehydrogenase 14 |
| *RDH16* | Retinol Dehydrogenase 16 |
| *RDH5* | Retinol Dehydrogenase 5 |
| *RDH8* | Retinol Dehydrogenase 8 |
| *RETSAT* | Retinol Saturase |
| *RHO* | Rhodopsin |
| *RXRA* | Retinoid X Receptor Alpha |
| *TSHR* | Thyroid Stimulating Hormone Receptor |
| **Group III. Genes of cytochrome P450 enzymes related to the retinol metabolism** | |
| *CYP19A1* | Cytochrome P450 family 19 subfamily A member 1 |
| *CYP1A1* | Cytochrome P450 family 1 subfamily A member 1 |
| *CYP1A2* | Cytochrome P450 family 1 subfamily A member 2 |
| *CYP1B1* | Cytochrome P450 family 1 subfamily B member 1 |
| *CYP26A1* | Cytochrome P450 family 26 subfamily A member 1 |
| *CYP26B1* | Cytochrome P450 family 26 subfamily B member 1 |
| *CYP2A13* | Cytochrome P450 family 2 subfamily A member 13 |
| *CYP2A6* | Cytochrome P450 family 2 subfamily A member 6 |
| *CYP2B6* | Cytochrome P450 family 2 subfamily B member 6 |
| *CYP2C18* | Cytochrome P450 family 2 subfamily C member 18 |
| *CYP2C19* | Cytochrome P450 family 2 subfamily C member 19 |
| *CYP2C8* | Cytochrome P450 family 2 subfamily C member 8 |
| *CYP2C9* | Cytochrome P450 family 2 subfamily C member 9 |
| *CYP2D6* | Cytochrome P450 family 2 subfamily D member 6 |
| *CYP2E1* | Cytochrome P450 family 2 subfamily E member 1 |
| *CYP2F1* | Cytochrome P450 family 2 subfamily F member 1 |
| *CYP2J2* | Cytochrome P450 family 2 subfamily J member 2 |
| *CYP2S1* | Cytochrome P450 family 2 subfamily S member 1 |
| *CYP2U1* | Cytochrome P450 family 2 subfamily U member 1 |
| *CYP3A4* | Cytochrome P450 family 3 subfamily A member 4 |
| *CYP3A43* | Cytochrome P450 family 3 subfamily A member 43 |
| *CYP3A5* | Cytochrome P450 family 3 subfamily A member 5 |
| *CYP3A7* | Cytochrome P450 family 3 subfamily A member 7 |
| *CYP4B1* | Cytochrome P450 family 4 subfamily B member 1 |
| *CYP4F11* | Cytochrome P450 family 4 subfamily F member 11 |
| *CYP4F12* | Cytochrome P450 family 4 subfamily F member 12 |
| *CYP4F8* | Cytochrome P450 family 4 subfamily F member 8 |
| *CYP4X1* | Cytochrome P450 family 4 subfamily X member 1 |
| *CYP4Z1* | Cytochrome P450 family 4 subfamily Z member 1 |
