# Supplementary material for: New associations of serum β‐carotene, lycopene, and zeaxanthin concentrations with NR1H3, APOB, RDH12, AND CYP genes
Source: Food Sci Nutr. 2022 Jan 8;10(3):763–71. doi: 10.1002/fsn3.2705 (PMC8907718; doi:10.1002/fsn3.2705)
Supplement: Supplementary file 2 — Table S2 [file FSN3-10-763-s001.docx]

Supplementary Table 2. Statistically significant SNPs associated with blood serum carotenoid concentrations.

| **Carotenoid** | **Chr^a^** | **Gene** | **SNP^b^** | **MA^c^** | **MAF^d^** | **MAF Q1^e^** | **MAF Q4^f^** | **Chi^2^** | **p-value** | **OR^g^ (95% CI^h^)** | **BONF^i^** | **EMP1^j^** | **EMP2^k^** |
| --- | --- | --- | --- | --- | --- | --- | --- | --- | --- | --- | --- | --- | --- |
| Zeaxanthin | 2p24.1 | *APOB* | rs550619 | G | 0.089 | 0.027 | 0.169 | 16.51 | 4.844×10^−5^ | 7.3 (2.5-21.9) | 0.03439 | 1.8×10^−5^ | 0.01612 |
|  | 11p11.2 | *NR1H3* | rs11039155 | A | 0.236 | 0.128 | 0.339 | 17.43 | 2.984×10^−5^ | 3.5 (1.9-6.4) | 0.01972 | 2.5×10^−5^ | 0.008414 |
|  |  |  | rs2279238 | A | 0.249 | 0.142 | 0.362 | 18.07 | 2.129×10^−5^ | 3.4 (1.9-6.1) | 0.01407 | 2.2×10^−5^ | 0.00557 |
| Lycopene | 14q24.1 | *RDH12* | rs756473 | G | 0.225 | 0.134 | 0.341 | 15.70 | 7.422×10^−5^ | 3.3 (1.8-6.2) | 0.04906 | 1.0×10^−4^ | 0.01865 |
| β-Carotene | 10q23.33 | *CYP2C9* | rs9332113 | C | 0.145 | 0.052 | 0.235 | 18.11 | 2.086×10^−5^ | 5.6 (2.4-13.2) | 0.00797 | 1.4×10^−5^ | 0.002792 |
|  |  |  | rs10509679 | A | 0.145 | 0.052 | 0.235 | 18.11 | 2.086×10^−5^ | 5.6 (2.4-13.2) | 0.00797 | 1.4×10^−5^ | 0.002792 |
|  |  |  | rs12569850 | G | 0.145 | 0.052 | 0.235 | 18.11 | 2.086×10^−5^ | 5.6 (2.4-13.2) | 0.00797 | 1.4×10^−5^ | 0.002792 |
|  |  |  | rs2298037 | A | 0.145 | 0.052 | 0.235 | 18.11 | 2.086×10^−5^ | 5.6 (2.4-13.2) | 0.00797 | 1.4×10^−5^ | 0.002792 |
|  |  | *CYP2C19* | rs6583954 | A | 0.136 | 0.046 | 0.212 | 16.34 | 5.294×10^−5^ | 5.7 (2.3-14.2) | 0.02022 | 3.2×10^−5^ | 0.006876 |
|  |  | *CYP2C18* | rs2860838 | C | 0.134 | 0.052 | 0.212 | 14.88 | 1.148×10^−4^ | 4.9 (2.1-11.6) | 0.04386 | 4.8×10^−5^ | 0.01496 |
|  |  |  | rs2296680 | A | 0.136 | 0.052 | 0.212 | 14.88 | 1.148×10^−4^ | 4.9 (2.1-11.6) | 0.04386 | 4.8×10^−5^ | 0.01496 |
|  |  |  | rs1926712 | G | 0.136 | 0.052 | 0.212 | 14.88 | 1.148×10^−4^ | 4.9 (2.1-11.6) | 0.04386 | 4.8×10^−5^ | 0.01496 |
|  |  |  | rs1326832 | G | 0.136 | 0.052 | 0.212 | 14.88 | 1.148×10^−4^ | 4.9 (2.1-11.6) | 0.04386 | 4.8×10^−5^ | 0.01496 |
|  |  |  | rs932809 | A | 0.136 | 0.052 | 0.212 | 14.88 | 1.148×10^−4^ | 4.9 (2.1-11.6) | 0.04386 | 4.8×10^−5^ | 0.01496 |
|  |  |  | rs2281890 | A | 0.136 | 0.052 | 0.212 | 14.88 | 1.148×10^−4^ | 4.9 (2.1-11.6) | 0.04386 | 4.8×10^−5^ | 0.01496 |
|  |  |  | rs1042194 | A | 0.136 | 0.052 | 0.212 | 14.88 | 1.148×10^−4^ | 4.9 (2.1-11.6) | 0.04386 | 4.8×10^−5^ | 0.01496 |
|  |  | *CYP2C19* | rs12768009 | A | 0.136 | 0.052 | 0.212 | 14.88 | 1.148×10^−4^ | 4.9 (2.1-11.6) | 0.04386 | 4.8×10^−5^ | 0.01496 |
|  |  |  | rs4244285 | A | 0.136 | 0.052 | 0.212 | 14.88 | 1.148×10^−4^ | 4.9 (2.1-11.6) | 0.04386 | 4.8×10^−5^ | 0.01496 |
|  |  |  | rs12571421 | G | 0.136 | 0.052 | 0.212 | 14.88 | 1.148×10^−4^ | 4.9 (2.1-11.6) | 0.04386 | 4.8×10^−5^ | 0.01496 |
|  |  |  | rs12767583 | A | 0.136 | 0.052 | 0.212 | 14.88 | 1.148×10^−4^ | 4.9 (2.1-11.6) | 0.04386 | 4.8×10^−5^ | 0.01496 |
|  |  |  | rs12772672 | G | 0.136 | 0.052 | 0.212 | 14.88 | 1.148×10^−4^ | 4.9 (2.1-11.6) | 0.04386 | 4.8×10^−5^ | 0.01496 |
|  |  |  | rs4641393 | A | 0.136 | 0.052 | 0.212 | 14.88 | 1.148×10^−4^ | 4.9 (2.1-11.6) | 0.04386 | 4.8×10^−5^ | 0.01496 |
|  |  |  | rs1853205 | C | 0.136 | 0.052 | 0.212 | 14.88 | 1.148×10^−4^ | 4.9 (2.1-11.6) | 0.04386 | 4.8×10^−5^ | 0.01496 |
|  |  |  | rs1322179 | A | 0.136 | 0.052 | 0.212 | 14.88 | 1.148×10^−4^ | 4.9 (2.1-11.6) | 0.04386 | 4.8×10^−5^ | 0.01496 |
|  |  |  | rs28399513 | A | 0.135 | 0.052 | 0.212 | 14.88 | 1.148×10^−4^ | 4.9 (2.1-11.6) | 0.04386 | 4.8×10^−5^ | 0.01496 |

^a^ – chromosome; ^b^ – single nucleotide polymorphism; ^c^ – minor allele; ^d^ – minor allele frequency in study population; ^e^ – quartile 1; ^f^ – quartile 4; ^g^ – odds ratio; ^h^ – confidence interval; ^i^ – Bonferroni single-step adjusted p-values, ^j^ – Empirical p-value (pointwise), ^k^ – Corrected empirical p-value (max(T) / family-wise), *APOB* - apolipoprotein B, *NR1H3* - nuclear receptor subfamily 1 group H member 3, *RDH12* - retinol dehydrogenase 12, *CYP2C9* - cytochrome P450 family 2 subfamily C member 9, *CYP2C19* - cytochrome P450 family 2 subfamily C member 19, *CYP2C18* - cytochrome P450 family 2 subfamily C member 18, *CYP2C19* - cytochrome P450 family 2 subfamily C member 1.
